# Voltammetric Electronic Tongue and Support Vector Machines for Identification of Selected Features in Mexican Coffee

**DOI:** 10.3390/s140917770

**Published:** 2014-09-24

**Authors:** Rocio Berenice Domínguez, Laura Moreno-Barón, Roberto Muñoz, Juan Manuel Gutiérrez

**Affiliations:** Bioelectronics Section, Electrical Engineering Department, CINVESTAV, 07360 Mexico D.F., Mexico; E-Mails: rdominguez@cinvestav.mx (R.B.D.); lauramorenob@gmail.com (L.M.-B.); rmunoz@cinvestav.mx (R.M.)

**Keywords:** coffee, electronic tongue, support vector machine, organic, geographical origin

## Abstract

This paper describes a new method based on a voltammetric electronic tongue (ET) for the recognition of distinctive features in coffee samples. An ET was directly applied to different samples from the main Mexican coffee regions without any pretreatment before the analysis. The resulting electrochemical information was modeled with two different mathematical tools, namely Linear Discriminant Analysis (LDA) and Support Vector Machines (SVM). Growing conditions (*i.e.*, organic or non-organic practices and altitude of crops) were considered for a first classification. LDA results showed an average discrimination rate of 88% ± 6.53% while SVM successfully accomplished an overall accuracy of 96.4% ± 3.50% for the same task. A second classification based on geographical origin of samples was carried out. Results showed an overall accuracy of 87.5% ± 7.79% for LDA and a superior performance of 97.5% ± 3.22% for SVM. Given the complexity of coffee samples, the high accuracy percentages achieved by ET coupled with SVM in both classification problems suggested a potential applicability of ET in the assessment of selected coffee features with a simpler and faster methodology along with a null sample pretreatment. In addition, the proposed method can be applied to authentication assessment while improving cost, time and accuracy of the general procedure.

## 1. Introduction

According to the International Coffee Organization (ICO), in the 2012 productive year, Mexico occupied the eighth position among the world coffee producers [1]. Mexican production is supported by approximately 15 regions of coffee growing, distinguished by geographical location, local microclimate and the particular cultivation procedures followed in each zone. To ensure coffee quality for final consumers, the Mexican government established different regulations for evaluation in accordance to international standards. In this sense, and given the importance of geographical location, Veracruz and Chiapas own a protected designation of origin (DO) [2,3]. Current regulation classifies as high grown coffee (HGC) to crops growing from 900 to 1200 m above sea level (m.a.s.l.) and as prime washed coffee (PW) to crops growing from 600 to 800 m.a.s.l. [4].

To ensure high standards in the coffee industry, extensive research to study key qualities in coffee has been lead. A considerable number of studies have been conducted to characterize the flavor [5], aroma [6,7], as well as to identify the chemical composition of coffee beans [8] and authenticity [9]. There are over 1000 different chemicals in coffee, including caffeine, sugars, polyphenols, chlorogenic acids, carbohydrates, amino acids and proteins, along with other chemical descriptors such as metals and minerals [10,11].

Different authors have used one or more of these elements as descriptors to determine specific features such as coffee authenticity, geographical origin, botanical variety and some characteristics related to growing and environmental conditions of crops [12–17]. Usually, the measuring is carried out by analytical methods, such as near-infrared spectroscopy (NIRS), liquid and gas chromatography (LC/GC), atomic absorption and emission spectrometry (AAS/AES), instrumental neutron activation analysis (INAA) and inductively coupled plasma optical emission spectrometry (ICP-OES) [18]. Commonly, the resulting information is processed by some chemometric methods in order to find patterns or features for samples discrimination. Principal component analysis (PCA), hierarchical cluster analysis (HCA), artificial neural networks (ANN) and self-organizing maps (SOM) are among the most applied methods [19,20].

However, in the present decade, artificial senses have been applied as alternative tools for traditional methodologies in food analysis [21]. These biomimetic systems, known as Electronic Tongues (ETs) and Electronic Noses (ENs), involve the use of a non-specific sensor array coupled with an appropriate chemometric tool for data processing [22,23]. ETs, in particular, have the ability to distinguish liquids of unknown composition without using any preliminary qualitative or quantitative information about the sample. Taking advantage of this peculiarity, different authors have used either potentiometric or voltammetric ETs for the identification and classification of beverages and foodstuffs [24]. However, the application of ETs in the analysis of coffee has been barely reported and only few articles have described the use of ETs for the identification or classification of a small set of different brands of ground roasted coffee [25,26].

The aim of this work is the development of a simpler, cheaper and faster methodology based on a voltammetric ET for the analysis of some features found in Mexican coffee beans. In general, this paper promotes an emerging methodology for the classification of coffee by type. It is important to note that this is a preliminary study. Today, in our laboratories, we are deepening the optimization of the electronic tongue presented in this paper, considering a vast array of samples, in order to extend the application of ET in the coffee industry. Even though Mexican coffee was taken as a model, the overall procedure can be extended for identification and authentication purposes of coffee samples from around the world.

## 2. Experimental Section

### 2.1. Coffees under Study

A total of 42 samples of ground roasted coffee (*Arabica type*) from some of the principal Mexican coffee regions were purchased from local producers. Thirty-four samples were selected from the two existing DO regions in Mexico (Veracruz and Chiapas). The remaining samples were chosen from a smaller coffee region without DO. In addition to geographic origin, features such as crops altitude and cultivation practices (*i.e.*, organic or non-organic production) were included in the selected samples.

According to crop altitude, 35 samples of the total set were identified as High Grown Coffee (HGC) while the last seven remaining samples were Prime Washed (PW) coffees. In relation to the cultivation practices, all PW samples and nine HGC samples were from organic crops. Therefore, to distinguish these samples, they were named as Organic Prime Washed coffee (OPW) and Organic Coffee (OC) respectively. In Table 1, characteristics for all samples are summarized. HGC refers to high grown coffee, OC depicts high grown coffee from organic crops and OPW represents organic coffee crops cultivated at lower altitudes.

### 2.2. Sensor Array

**Figure 1.**
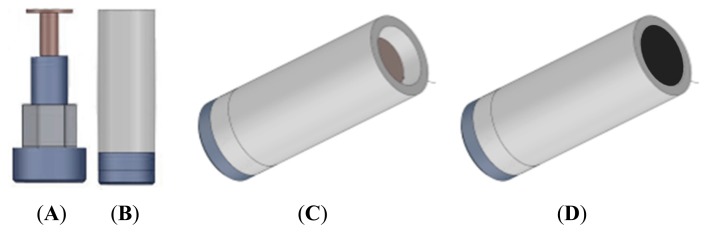
Graphite-epoxy composite electrodes construction scheme. (**A**) Copper disk soldered to the electrical connector; (**B**) Electrical section assembled into the PVC tube; (**C**) Body sensor ready to be filled with the composite paste; (**D**) Final appearance after hardening and polishing.

An array of six graphite-epoxy voltammetric sensors, made with different modifiers added to the bulk mixture, was selected according to previous studies with foodstuffs [27–29]. For the standard graphite-epoxy voltammetric sensor, a bulk mixture of 20 μm particle size graphite powder (Sigma Aldrich) and Epotek H77 resin and hardener (both from Epoxy Technology, Billerica, MA, USA) was used. For the five remaining voltammetric sensors, different modifiers such as platinum and gold nanoparticles (Sigma-Aldrich, St. Louis, MI, USA), cobalt II phthalocyanine (Sigma-Aldrich, St. Louis, MI, USA) and conducting polymers as polyaniline (Sigma-Aldrich, St. Louis, MI, USA) and polypirrole (Sigma-Aldrich, St. Louis, MI, USA) were added to the bulk mixture. Afterwards, each composite was manually homogenized for 60 min before filling a PVC body (see Figure 1). Then, the sensors were allowed to harden for 7 days at 40 °C. Finally, each sensor surface was polished with different sandpapers of decreasing grain size, in order to obtain a homogeneous working electrode area of 28 mm^2^. The measurement cell was formed by the six voltammetric sensor array, a reference double junction Ag/AgCl electrode (Thermo Orion 900200) and a commercial platinum counter electrode (Model 52–67, Crison Instruments).

### 2.3. Sample Preparation

Standard procedure for sample preparation was based on current Mexican DO norms and local producer specifications [2–4]. Briefly, 8 g of ground roasted coffee were collected from a newly open packet. Meanwhile, bottled water (Bonafont S.A. de C.V, Mexico) was heated until boiling point. Coffee ground was placed in a coffee machine filter while 50 mL of boiled water were added. Finally, the resulting infusion was stored until room temperature (25 °C) was achieved.

### 2.4. Electrochemical Technique and Procedure

Before performing the measurements with coffee samples, the built electrodes were cycled for 3–5 times in distilled water until they reached a stable response. Cyclic voltammetry measurements were made using a 6-channel AUTOLAB/PGSTAT20 (Ecochemie, The Netherlands). All measurements were carried out at room temperature (25 °C) under quiescent condition and without any sample pretreatment. For measurements, scan rate was fixed at 0.1 V·s^−1^ with a step potential of 9 mV. Potential sweep was set in the range of −1.7–1.5 V *vs*. Ag/AgCl. All prepared infusions were measured according to these set up parameters. In addition, with the purpose of normalizing all registers, a blank register (only bottled water) was recorded before each sample measurement. All experiments were carried out without performing any physical surface regeneration of the working electrodes. In order to prevent the accumulative effect of impurities on the working electrode surfaces, an electrochemical cleaning stage was performed between each measurement applying a conditioning potential of +1.8 V for 40 s after each experiment, in a cell containing 10 mL of distilled water.

### 2.5. Data Processing

Data processing and modelling was done by specific routines written by the authors in MATLAB 2012b (MathWorks, Natick, MA, USA), based on already preprogrammed standard functions using Statistics Toolbox (v8.1) and the free software LibSVM 3.18 [30]. From the five scans performed in each coffee sample determination, only the last one was used as input data. The whole cyclic voltammograms from the six sensor array were included in the different data processing stages.

Firstly, the available raw voltammetric data was preprocessed considering a blank correction, centering and standardization (*i.e.*, standard deviation = 1 and mean = 0) in order to build a preliminary recognition model using Principal Component Analysis (PCA). Secondly, the same raw data was blank corrected and normalized prior to be modeled with two classification methods namely Linear Discriminant Analysis (LDA) and Support Vector Machines (SVM). For training convenience, the preprocessed voltammetric was randomly split into two subsets, 70% of the total information was taken for training and the rest for testing. Given that those are supervised methods, the classification success (was evaluated using a *k*-fold (*k* = 3) cross-validation technique selecting the test set each time at random from the total set of samples. A total of 10 replicates were considered to calculate the average accuracy, sensitivity and specificity of the models.

Besides comparing LDA and SVM models the goal was to identify the effect of using mathematical models of linear and nonlinear characteristics. As is well known, LDA is one of the most widely used classification procedures, which has proven success in many applications. This method maximizes between class variability relative to within-class variability. For LDA modeling, the data set is projected onto a new dimensional space based on a target vector of class labels. Unlike PCA, where dimensionality reduction is only based on maximum data variance, LDA seeks for dimensionality reduction but keeps the class discriminatory information provided by class label vector. On the new projected space, each sample was assigned to its corresponding group according to Euclidean distance from centroid class [31]. Once trained, LDA model was validated against a test group, where Euclidean distance was calculated as well to assign new samples with the predicted group.

In counterpart, SVM modeling represent a new approach to pattern classification that has attracted a lot of attention in many real-world applications ranging from data mining, chemistry and biotechnology [32,33]. Its principle comes from the framework of the statistical learning theory, which is appropriate for approaching classification and regression problems [34,35]. Probably, the major advantage of SVM is related to their global and unique solution avoiding the multiple local minima problem of models such as artificial neural networks. In addition, the final complexity of SVM models does not depend on the dimensionality of the input space. SVM models are also less susceptible to the well-known problem of overfitting, since they operate on the induction principle of structural risk minimization (which minimizes an upper bound on the generalization error). In this work, SVM discrimination based on linear and radial basis function (RBF) kernels was studied. Before validation, the parameters in both kernels were optimized with a training data set.

## 3. Results and Discussion

### 3.1. Exploratory Data Analysis

**Figure 2.**
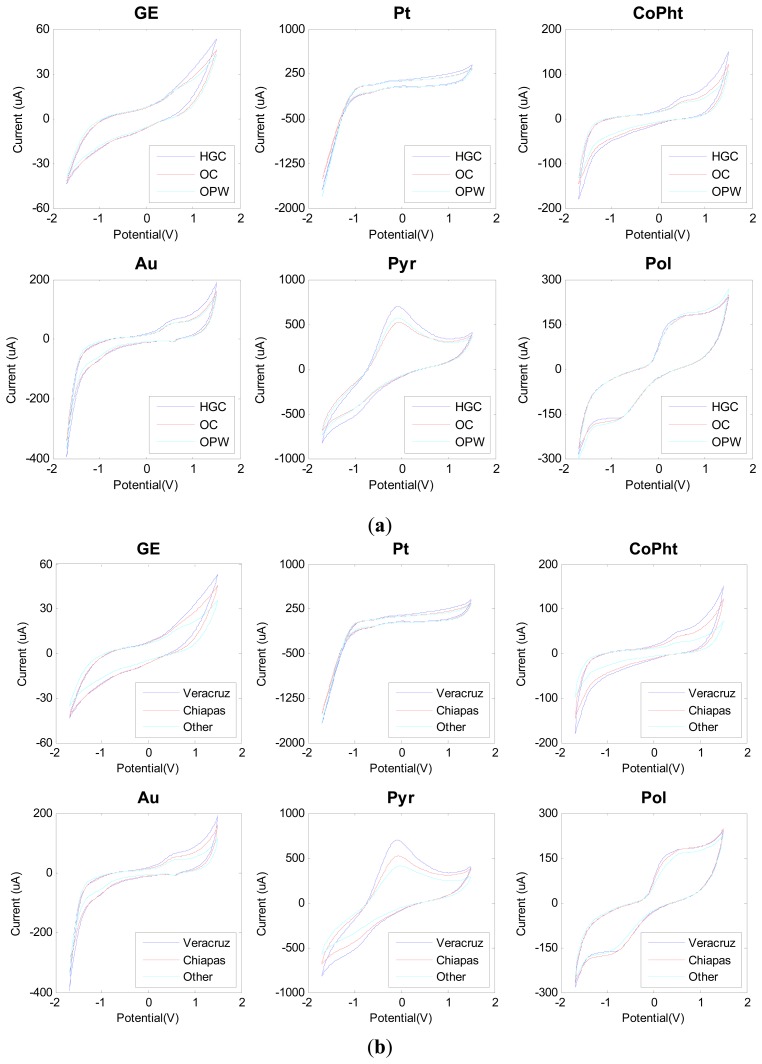
Recorded voltammograms from Graphite Epoxy (GE), Platinum nanoparticle (Pt), Cobalt II phthalocyanine (CoPht), Gold nanoparticle (Au), Polypirrole (Pyr) and Polyaniline (Pol) sensors for three different coffee samples. Voltammograms for growing conditions are shown in (**a**) and voltammograms for geographical origin can be observed in (**b**).

Figure 2 shows the measured voltammograms with distinctive signals for each sensor. Catalytic oxidative signals seem to be originated from the metal nanopaticle modified sensors, which may be due to a catalytic oxidation of saccharides and/or polyphenols on the sensor surface. Similarly, sensors modified with conducting polymers and phthalocyanines bring new information with completely different waveforms and distinct redox peaks.

Similarly, the reader is referred to the information presented in the supplementary material to visualize a blank signal of each sensor. Different Figures S1–S6 show the significance of the voltammograms in front of the blank. It may be noted that the measured voltammograms exhibit variations not only in current intensities, but also in shape.

**Figure 3.**
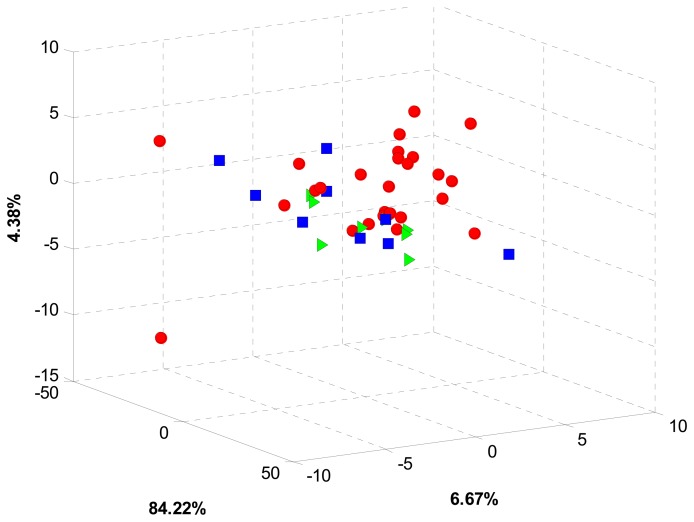
Coffee data representation by its three first principal components. Red dots represent HGC, blue squares represent samples OC coffee and green triangles represent samples OPW coffee.

Given the clear response variability of sensors, the first processing stage was done using PCA in order to find some sort of pattern in sensor data. For this stage, a preprocessing stage covering blank correction, centering and standardization was applied to the whole voltammetric data. Multidimensional information coming from the sensor array was then arranged into a matricized array of dimension 42 × 4272 (samples × stacked measurements from the six sensors). Considering the differences in geographical origin and growing conditions, a grouping trend, according to some of these two features, was expected. However, even though the cumulated total variance was 95.27%, PCA analysis did not show any significant pattern in data. Moreover, a strong scattering trend was observed for all the coffee samples as can be observed in Figure 3.

Despite the high cumulated variance, the low discrimination achieved by PCA analysis can be explained by the nonlinear nature of sensor data. Also, dimensionality reduction based exclusively on variance can dismiss some minor features found in voltammograms, which can potentially contribute to the cross sensitivities required for an ET approach. Since no trend was observed with PCA, a supervised approach based on LDA and SVM was pursued instead. In this sense, data was arranged according to two major characteristics: growing conditions and geographical origin.

### 3.2. Growing Classification of Coffee Samples

Voltammetric data was analyzed for a classification based exclusively on coffee growing conditions (*i.e*., cultivation procedure and altitude of crops). Growing conditions from Table 1 described a set of three different classes namely HGC, OC and OPW coffee samples. To preserve the richness of the analytical signals, the whole records were used without feature extraction or previous dimensionality reduction. Only blank correction plus normalization were applied for preprocessing data in each model. The supervised methods LDA and SVM were used to attempt to correlate the overall voltammetric signals with one of the three described classes; each model showed distinctive performance according with their operational characteristics. From the available information, different classifiers were built using LDA and SVM, in order to compare their classification performance.

**Figure 4.**
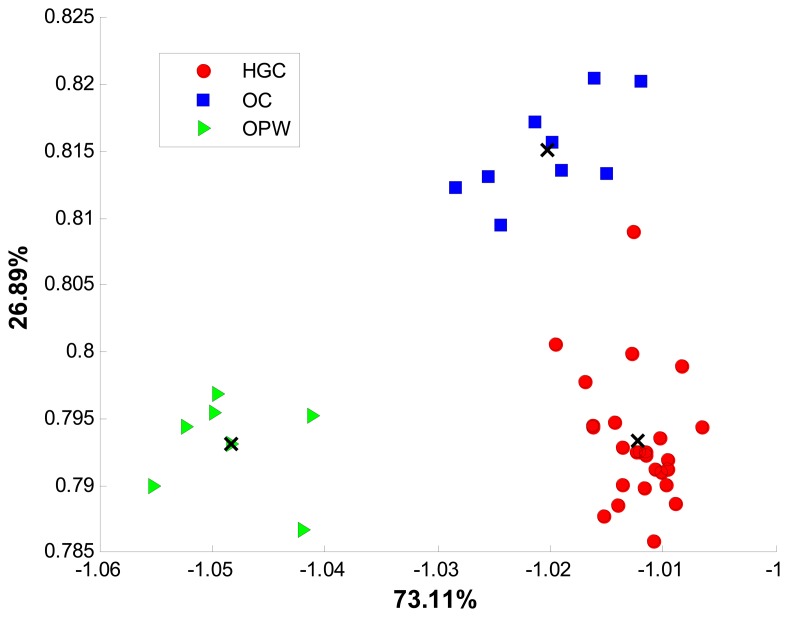
LDA classification according to growing conditions.

Figure 4 shows the discrimination achieved for all samples with the new bidimensional space of two discriminant functions.

**Figure 5.**
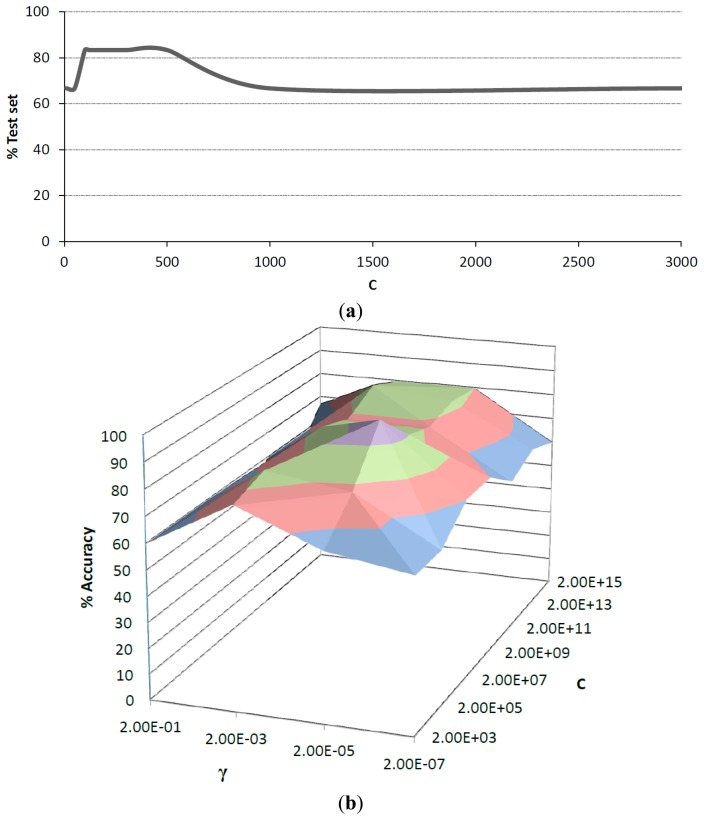
(**a**) Cost parameter evaluation for linear kernel; (**b**) RBF kernel surface response for cost parameter and kernel coefficient.

As mentioned earlier, SVM discrimination was based on linear and radial basis function (RBF) kernels. Before the validation stage, the parameters in both kernels were optimized. For linear kernel, the cost parameter *c* was evaluated in the range of 0–3000. After training, results from test set showed a maximum accuracy of 83.3% in the range of 100–500. Values outside this range exhibited strong overfitting trend, which can be observed in Figure 5a. For RBF kernel the cost parameter *c* and the kernel coefficient γ, were studied in the range of 2 × 10^3^ to 2 × 10^15^ for *c* and 2 × 10^−1^ to 2 × 10^−7^ for γ. It was observed that γ values in the upper and lower limits of this range resulted in overfitting, while middle values tend to be more accurate. Figure 5b shows the surface obtained for the different *c* and γ values. A maximum percentage of accuracy for RBF kernel was observed with *c* equal to 2 × 10^7^ and γ equal to 2 × 10^−5^, which was chosen over the linear kernel.

**Figure 6.**
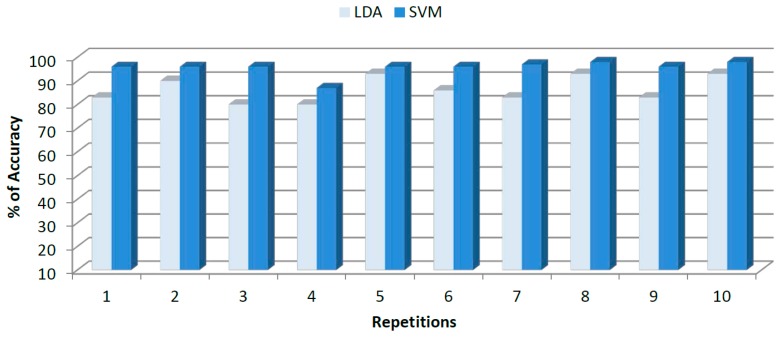
Final results obtained for growing conditions classification after *k*-fold cross-validation data using LDA and SVM models.

The results of accuracy (classification rate) for LDA and SVM models after ten replications of *k*-fold cross validation are shown in Figure 6. As can be observed, LDA models displayed inconsistent accuracy results, ranging from 80% in the worst case to 96% in the best case. However, for the same data SVM models showed a constant trend with high accuracy percentage ranging from 93.75% in the worst case and 100% in the best case. For growing classification the average percentage of accuracy for LDA was 88% ± 6.53% and 96.4% ± 3.50% for SVM. From both results, accuracy percentage and total deviation, a superior SVM performance as compared to LDA can be noted.

In addition to accuracy, sensitivity (rate of correctly classified objects for each class) and specificity (rate of correctly rejected objects for each class) of the two classifiers were calculated. The average results for each class after 10 replicates are presented in Table 2 along with the calculated deviations and the overall results.

From Table 2, some results can be highlighted. The high obtained rates in sensitivity and specificity percentages exhibited by LDA and SVM in the third class established a clear distinction between coffee samples cultivated at high altitudes (HGC and OC classes) and those cultivated at sea level (*i.e.*, OPW). This attribute identified by the ET could be associated with the quality of Mexican coffee; nevertheless this fact should be confirmed in further studies. HGC and OC classes were cultivated on high altitude crops; however, a high discrimination rate between samples cultivated with organic practices and those cultivated with standard practices was successfully achieved by SVM and with a minor success by LDA. The sensitivity and specificity showed by SVM in HGC class suggested that almost all samples from this group were correctly classified and no OC or OPW samples were misclassified as HGC. All the OC class members were correctly classified and only a minor percentage of total set was misclassified as a member of this group. However, although both classifiers adequately distinguish different classes, only SVM models allowed the maximal accuracy.

Even a similar discrimination between organic and non-organic samples was previously reported, the resulted achieved by voltammetric ET is remarkable because of the easier instrumental method, null sample pretreatment, the inclusion of samples from different geographical regions and the high accurate accomplished results [14].

### 3.3. Geographical Classification of Ground Roasted Coffee

Beyond the discrimination of the growing procedure of coffee samples, the other objective was to evaluate ET capability to perform a geographical classification of ground roasted samples. Since the coffee samples identified as HGC constitute the largest class, only the geographical origin classification for these samples was considered. To ensure coffee origin, HGC samples were purchased from local producers in each Mexican region. First considered classes were Veracruz and Chiapas because of their protected DO for coffee. In a third class, samples from Oaxaca (without DO) were grouped together.

**Figure 7.**
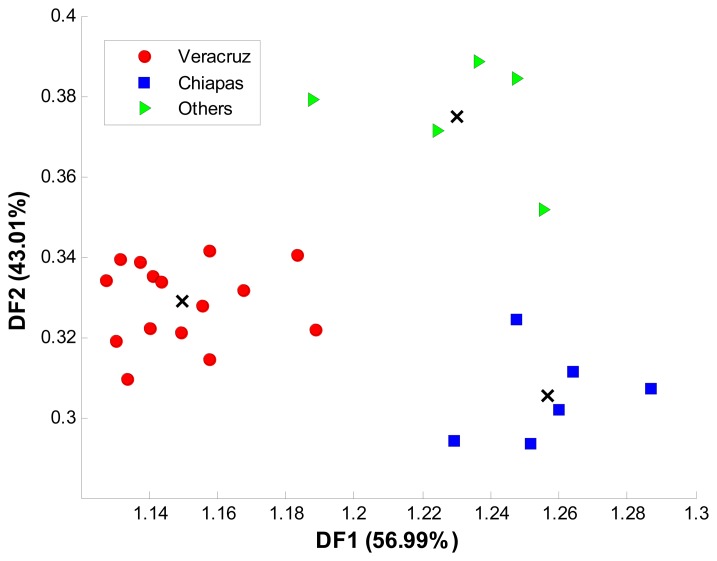
Class distribution achieved by LDA for geographical origin of samples.

Recorded voltammograms from the 26 available coffee samples were processed with LDA and SVM using the same optimization procedure described in Section 3.2. Final SVM values of *c* equal to 2 × 10^7^ and γ equal to 2 × 10^−1^ were chosen. The two models were tested *k*-fold cross validation and 10 replicates. Average accuracy, sensibility and sensitivity were calculated for each model. In Figure 7, the class distribution in the two dimensional space obtained by LDA is showed. As in growing conditions’ classification, Euclidean distance was used to predict the selected group for a given sample.

**Figure 8.**
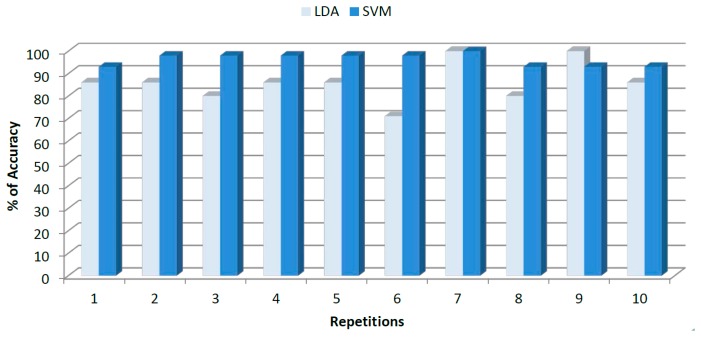
Final results obtained for geographical origin classification after *k*-fold cross-validation data using LDA and SVM models.

Finally, Figure 8 shows the accuracy achieved by LDA and SVM for cross validation data after 10 replicates.

Average accuracy achieved by LDA was 87.5% ± 7.79%, while SVM showed a superior performance of 97.5% ± 3.22%. The higher deviation of ±7.79% for LDA can be noted on the inconsistent trend showed by Figure 8. Sensitivities and specificities for each class along with the overall performance are shown in Table 3.

Because of the existing DO for Veracruz and Chiapas, the results obtained by SVM are remarkable for the geographical origin classification problem. Firstly, voltammetric ET and SVM clearly established a distinction between coffee samples from different geographical regions. From the three classes, discrimination was expected for Veracruz and Chiapas groups because of the existing DO for coffee samples. Even legislation established a quality control based on sophisticated instrumental methods, the proposed voltammetric ET plus SVM lead to results higher than 98% of accuracy for this data set. Specificities accomplished by SVM also shown that Veracruz samples reached an outstanding performance of 100%, while for Chiapas and the outlier region there was a minor misclassification. Even some early studies approached the geographical origin classification of coffee samples based on chemical profile, mineral content or metabolic markers with promising results, this is the first time geographical origin of coffee samples is successfully classified by a voltammetric sensor array and a chemometric tool. As compared with other studies, the results presented in this work showed an improvement in accuracy, plus the advantage of an easier, inexpensive and fast methodology [36]. Moreover, even results covered samples from at least three different Mexican regions, the methodology can be potentially extended to a higher number of worldwide regions.

## 4. Conclusions

An instrumental method based on voltammetric ET and SVM for the analysis of distinctive coffee qualities was presented. For classification, samples from the principal Mexican coffee regions and samples with DO were included in the data set. Discriminatory capability based on key features such as growing conditions and geographical origin was performed. Two mathematical models, namely LDA and SVM were applied for classification of voltammetric data. After optimization, best results for the two proposed classifications were achieved with SVM using a RBF kernel. Successful discrimination between samples cultivated with different conditions (*i.e.*, altitude, organic and non-organic coffee practices) was achieved with an overall accuracy of 96.4% ± 3.50%, while for geographical origin the accomplished accuracy was 97.5% ± 3.22%. Data modeling showed the importance of inclusion of several pattern recognition methods based on linear and nonlinear trends for the analysis of complex data coming from sensor arrays. The inclusion of nonlinear modeling allowed the discrimination of coffee samples cultivated at different altitudes, the distinction between organic and non-organic coffee, as well as the recognition of samples from principal Mexican coffee regions. All these are considered important features to assess coffee quality. The presented results pointed out the potential of application of an ET as an easy, fast and inexpensive method for coffee analysis or authentication assessment in the quality control industry.

## Supplementary Material



## Figures and Tables

**Table 1.** Characteristics for the 42 coffee samples data set.

**Geographical Region**	**Brand**	**Growing Conditions**
Veracruz	*Café Xico*	*HGC*
	*Los Portales*	*HGC*
	*Avelino Tueste Reserva*	*HGC*
	*Texolo*	*HGC*
	*D’La Finca*	*HGC*
	*Blasón Coatepec*	*HGC*
	*Bordi*	*HGC*
	*Baxtla*	*HGC*
	*Avelino Tueste Exprés*	*HGC*
	*Café Junco*	*HGC*
	*Café Córdoba*	*HGC*
	*Avelino Tueste Exprés*	*HGC*
	*Punta de Oro*	*HGC*
	*La Onza Gourmet*	*HGC*
	*Moretto*	*HGC*
	*La Misión*	*OC*
	*Buena Ventura Premium*	*OC*
	*Buena Ventura Gourmet*	*OC*
	*Abaxomol*	*OC*
	*Sierra de los Tuxtlas*	*OPW*
	*Etrusca Coatepec*	*OPW*
	*Don Pepe*	*OPW*
Chiapas	*Soconusco*	*HGC*
	*Blasón Jaltenango*	*HGC*
	*Nacional Café David*	*HGC*
	*Caracol*	*HGC*
	*Prensado Francés*	*HGC*
	*Mamá José*	*HGC*
	*Morteador*	*OC*
	*Oro Maya*	*OC*
	*Mulantic*	*OC*
	*BioStricto*	*OC*
	*Etrusca Soconusco*	*OPW*
	*Yajalón*	*OPW*

Others		
	*Blasón Pluma*	*HGC*
	*Gila Gourmet*	*HGC*
	*Gourmet Urrios*	*HGC*
	*Sierra de Oaxaca*	*HGC*
	*Tres Oros*	*HGC*
	*Blasón artesanal*	*OC*
	*Uciri*	*OPW*
	*Rincón de Ixtlán*	*OPW*

**Table 2.** Sensitivity and specificity for growing conditions classification after cross-validation data.

**Class**	**LDA**		**SVM**	
	
	**Sensitivity (%)**	**Specificity (%)**	**Sensitivity (%)**	**Specificity (%)**
HGC	85 ± 10.29	96.66 ± 7.5	97.5 ± 6.03	98.88 ± 3.51
OC	88 ± 13.98	96.26 ± 6.6	90 ± 10.5	98 ± 4.38
OPW	100 ± 0	90.99 ± 7.1	100 ± 0	98.09 ± 2.46
Overall	91 ± 8.09	94.63 ± 7.06	95.83 ± 5.51	98.32 ± 3.45

**Table 3.** Sensitivity and specificity for geographical origin classification for cross-validation data.

**Class**	**LDA**		**SVM**	
	
	**Sensitivity (%)**	**Specificity (%)**	**Sensitivity (%)**	**Specificity (%)**
Veracruz	81.10 ± 12.88	95.71 ± 6.90	94.44 ± 7.86	100 ± 0
Chiapas	100 ± 0	91.43 ± 8.79	100 ± 0	97.49 ± 4.02
Others	89.99 ± 16.10	94.55 ± 9.64	100 ± 0	98.46 ± 4.86
Overall	90.36 ± 9.66	93.89 ± 8.44	98.14 ± 2.62	98.65 ± 2.96
