# Supplementary material for: Voltammetric Electronic Tongue and Support Vector Machines for Identification of Selected Features in Mexican Coffee
Source: Sensors (Basel). 2014 Sep 24;14(9):17770–85. doi: 10.3390/s140917770 (PMC4208248; doi:10.3390/s140917770)
Supplement: Supplementary file 1 [file sensors-14-17770-s001.pdf]

OPEN ACCESS

sensors

ISSN 1424-8220

www.mdpi.com/journal/sensors

Supplementary Information

Voltammetric Electronic Tongue and Support Vector Machines for Identification of Selected Features in Mexican Coffee. *Sensors* 2014, *14*, 17770-17785

Rocio Berenice Domínguez, Laura Moreno-Barón, Roberto Muñoz and Juan Manuel Gutiérrez *

Bioelectronics Section, Electrical Engineering Department, CINVESTAV, 07360, Mexico D.F., Mexico; E-Mails: rdominguez@cinvestav.mx (R.B.D.); lauramorenob@gmail.com (L.M.B.); rmunoz@cinvestav.mx (R.M.)

* Author to whom correspondence should be addressed; E-Mail: mgutierrez@cinvestav.mx; Tel.: +52-55-5747-3800; Fax: +52-55-5747-3981

Supplementary Data

This supplementary provides detailed information about the voltammetric responses measured of coffee samples (*i.e.*, High Grown Coffee (HGC), Organic Prime Washed coffee (OPW) and Organic Coffee (OC)) considered in this work. Different figures included show the significance of the voltammograms in front of the blank signal. It may be noted that the measured voltammograms exhibit variations not only in current intensities, but also in shape. Such patterns in voltammetric signals are a clear evidence of the rich information that contain.

Figure S1. Graphite-epoxy voltammetric sensor and measured responses for different coffee samples and blank.

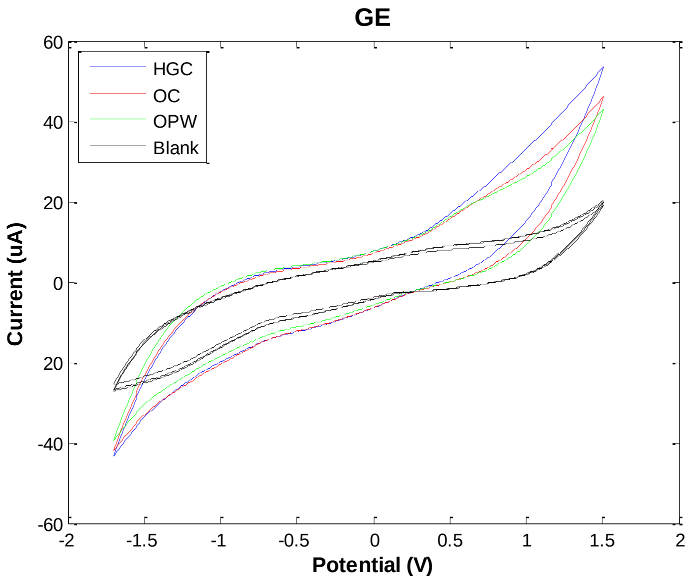

Figure S2. Platinum nanoparticle voltammetric sensor and measured responses for different coffee samples and blank.

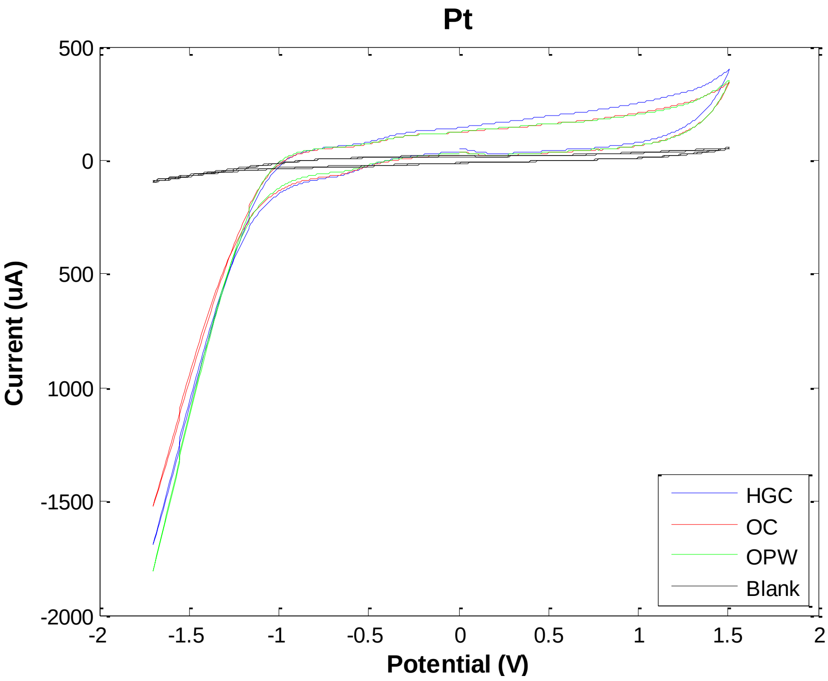

Figure S3. Gold nanoparticle voltammetric sensor and measured responses for different coffee samples and blank.

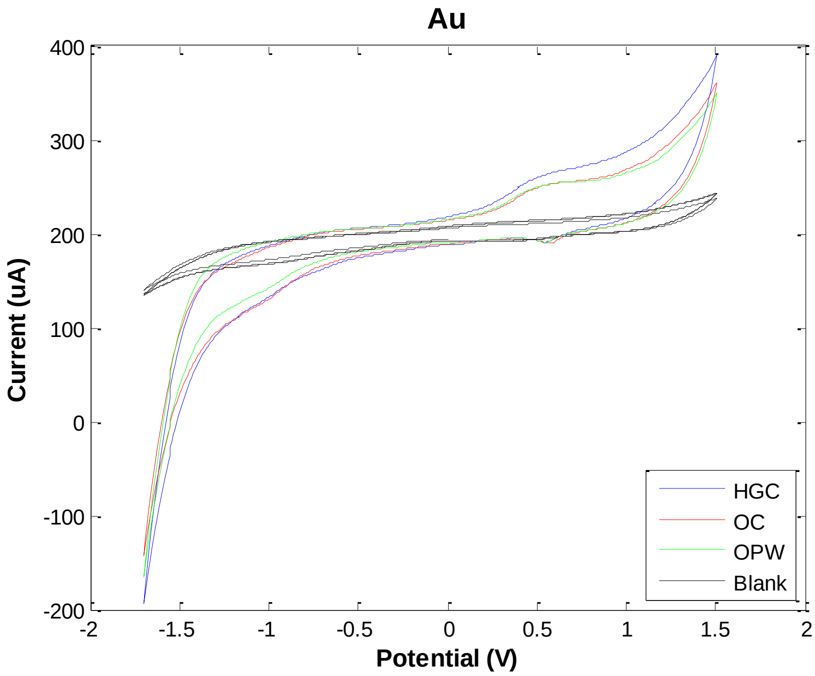

Figure S4. Cobalt II phthalocyanine voltammetric sensor and measured responses for different coffee samples and blank.

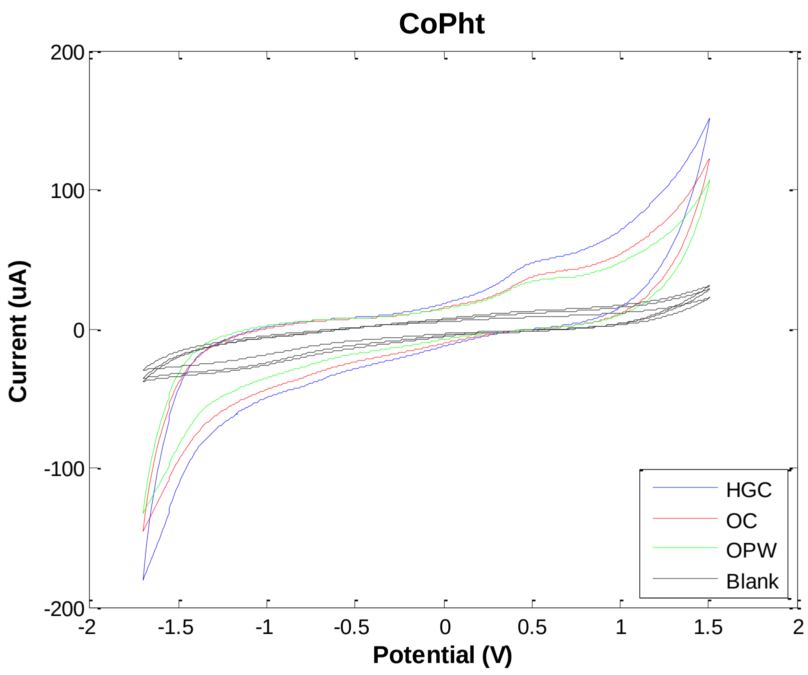

Figure S5. Polypirrole voltammetric sensor and measured responses for different coffee samples and blank.

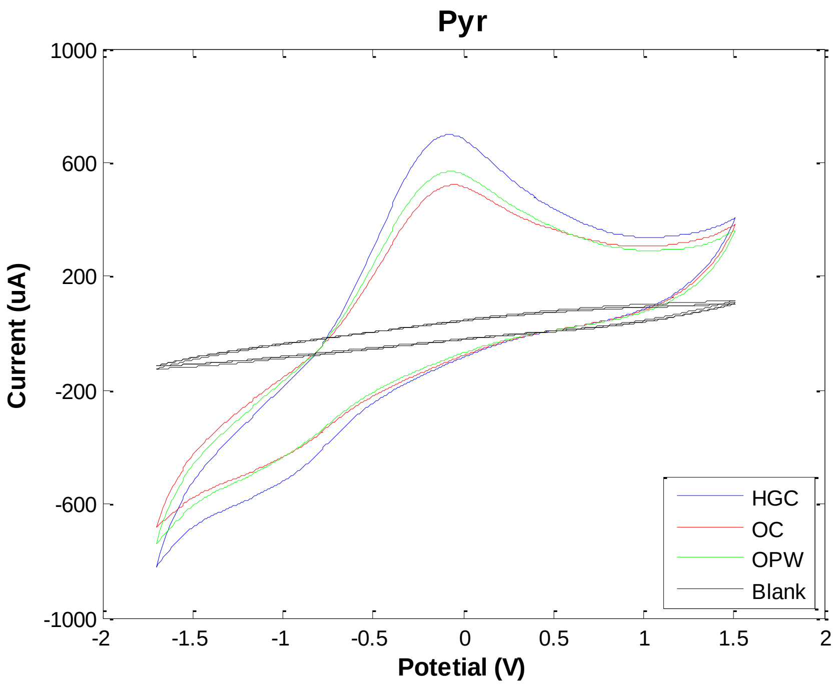

Figure S6. Polyaniline voltammetric sensor and measured responses for different coffee samples and blank.

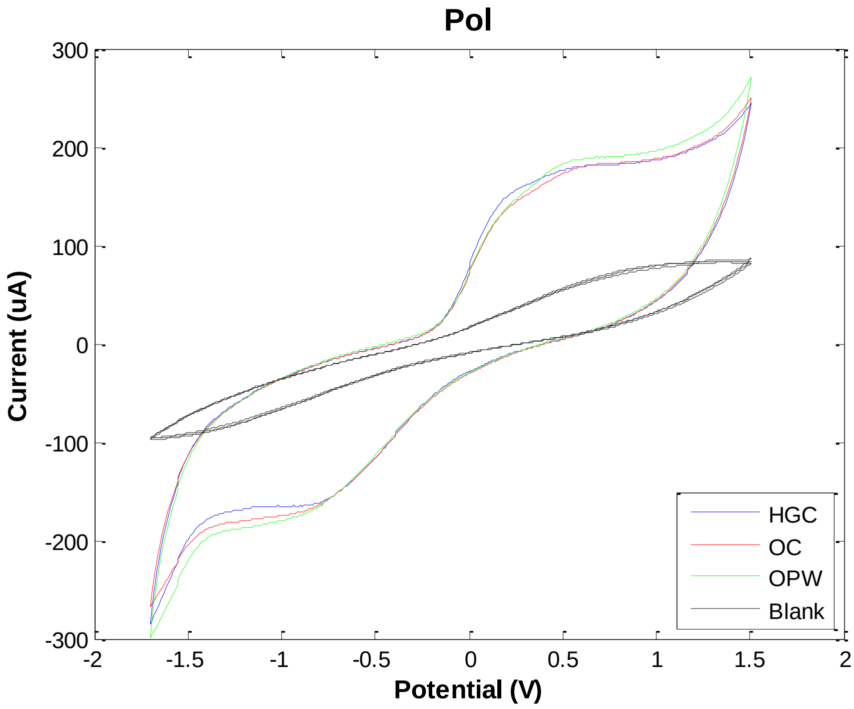

© 2014 by the authors; licensee MDPI, Basel, Switzerland. This article is an open access article distributed under the terms and conditions of the Creative Commons Attribution license (http://creativecommons.org/licenses/by/3.0/).
